# Endobronchial valves for severe air leak in critically ill children with necrotizing pneumonia requiring extracorporeal membrane oxygenation

**DOI:** 10.1177/02676591251365419

**Published:** 2025-08-11

**Authors:** Alex J. Katz, Bhavesh M. Patel, Vanessa M. Mazandi, Lauren M.C. Grant, Aoife Corcoran, Pelton Phinizy, Antoinette Wannes Daou, Garrett Keim, Paula M. Magee, Myron Allukian, Todd J. Kilbaugh, Sin Tran, Adam S. Himebauch, Joseph Piccione, Kumaran Senthil

**Affiliations:** 1 Division of Pulmonary and Sleep Medicine, Department of Pediatrics, 6567 Children’s Hospital of Philadelphia, Philadelphia, PA, USA; 2 Division of Pediatric Critical Care Medicine, Department of Anesthesiology and Critical Care Medicine, 6567 Children’s Hospital of Philadelphia, Philadelphia, PA, USA; 3 Perelman School of Medicine at the University of Pennsylvania, Philadelphia, PA, USA; 4 Division of General Anesthesiology, Department of Anesthesiology and Critical Care Medicine, Children's Hospital of Philadelphia, Philadelphia PA, USA.; 5 Leonard Davis Institute of Health Economics, University of Pennsylvania, Philadelphia, PA, USA; 6 Division of Pediatric General, Thoracic, and Fetal Surgery, Department of Surgery, Children’s Hospital of Philadelphia, Philadelphia, PA, USA; 7 Department of Nursing and Clinical Care Services, 6567 Children’s Hospital of Philadelphia, Philadelphia, PA, USA

**Keywords:** pneumothorax, extracorporeal membrane oxygenation, pneumonia, necrotizing, critical illness, intensive care units, pediatric

## Abstract

**Introduction:**

Air leak is a common complication of necrotizing pneumonia in critically ill children. Severe air leaks impact effective oxygenation and ventilation, oftentimes necessitating multiple thoracostomy tubes and extracorporeal support. Endobronchial valves (EBV) are a minimally invasive intervention to control air leak that may expedite de-escalation of care for critically ill children with necrotizing pneumonia.

**Methods:**

A retrospective case series was conducted on patients at the Children's Hospital of Philadelphia who were hospitalized in the pediatric intensive care unit, required extracorporeal membrane oxygenation (ECMO), and underwent placement of one or more EBVs for air leak from July 2023 through August 2024.

**Results:**

Six patients, median age 12 years (range 18 months to 18 years), were hospitalized for necrotizing pneumonia complicated by severe air leak and required ECMO. The most common etiology was a viral infection with bacterial co-infection. The median number of EBVs placed per patient was five. The median total time on ECMO was 10 days, with a median duration of 3.5 days after valve placement. The median duration of antibiotic therapy was 47 days (range 24 to 126 days). One patient had a contralateral pneumothorax after valve placement, and another died due to progression of multiorgan failure that began before EBV placement. The five surviving patients were discharged from the hospital, weaned from all respiratory support, and their valves were removed without complication.

**Conclusion:**

Endobronchial valves are a feasible intervention for severe air leak in critically ill children with necrotizing pneumonia and may expedite liberation from ECMO.

## Introduction

Among critically ill children with necrotizing pneumonia, air leak is a common complication, occurring in up to 25% of patients and is independently associated with mortality.^1–3^ Conservative management of air leak includes thoracostomy tube drainage with a period of clinical observation to allow for spontaneous resolution. Air leak may interfere with oxygenation and ventilation despite thoracostomy tube drainage, sometimes necessitating extracorporeal membrane oxygenation (ECMO) support.^3–5^ Even without accounting for the additional morbidity of air leak, children who require ECMO for pneumonia have an estimated mortality of 39%.^6^

For persistent air leaks, defined as those lasting longer than 5-7 days, additional treatment options may include: an external one-way valve, portable suction connected to a thoracostomy tube, pleurodesis via sclerosing agents, mechanical abrasion, autologous blood patch administration, surgical lobectomy in extreme cases.^7,8^ Finally, endobronchial valves (EBV) have emerged as a promising minimally invasive intervention for air leaks.

EBVs have been used for air leak following lobectomy or pneumonectomy, emphysema, trauma, and pneumonia, and have more recently been approved for bronchoscopic lung volume reduction in emphysema.^9–17^ In adults undergoing EBV placement for air leak, over 90% show reduction or complete resolution of the air leak.^18,19^ More recently, EBV placement has been used to treat air leaks in children, with early case reports suggesting this technique is both safe and effective despite its off-label use.^5,20–24^ However, there is limited data available on the feasibility of EBV placement in severe air leak requiring mechanical ventilation and ECMO in children with necrotizing pneumonia.

We present our single-center experience with placement of EBVs in six patients with severe air leak in the setting of necrotizing pneumonia with associated acute respiratory failure requiring mechanical ventilation and ECMO. Although air leaks are often qualitatively graded by the Cerfolio classification, we defined a severe air leak as a continuous air leak that interfered significantly with oxygenation and ventilation as to necessitate mechanical ventilation.^25^ Our objective was to assess the feasibility of placing endobronchial valves for severe air leak in critically ill children with necrotizing pneumonia.

## Methods

This is a retrospective case series of all patients at the Children's Hospital of Philadelphia who were hospitalized in the pediatric intensive care unit (PICU), required ECMO, and underwent placement of one or more EBVs for severe air leak from July 2023 through August 2024. The study was deemed exempt from Institutional Review Board oversight (IRB 24-022518).

### Valve Placement

All children received their EBVs under sedation and neuromuscular blockade in the PICU. The Olympus Spiration Valve System (Olympus Corporation, Japan) was used. A flexible bronchoscope with sequential balloon occlusion was used to isolate the location of the air leak. Due to the large working channel necessary for the 2.6 mm deployment catheter, the EBVs were placed with a large disposable 5.8 mm flexible bronchoscope through a laryngeal mask airway or 7.0 mm or larger endotracheal tube. If the patient was too small to accommodate a 7.0 mm or larger endotracheal tube and it was not feasible to place a laryngeal mask airway, a side-by-side approach was used with the 2.6 mm deployment catheter running externally to a 2.2 mm flexible bronchoscope. Bedside evaluation of the thoracostomy tube apparatus and serial imaging were used to assess resolution of the air leak. Heparin anticoagulation necessary for ECMO was not interrupted for the bronchoscopy and valve placement. Primary teams were instructed to refrain from using intrapulmonary percussive ventilation while EBVs were in place. EBVs were removed at least 6 weeks following resolution of the air leak, based on the patient’s clinical status and resolution of underlying disease process. After valve removal, a chest X-ray was obtained to evaluate for recurrence of pneumothorax and patients were monitored overnight in the hospital prior to discharge.

### Data abstraction and analysis

The electronic medical record was abstracted for demographic characteristics, any procedural interventions including intubation, thoracostomy tube placement, ECMO cannulation, and EBV placement, antibiotic regimens, outcomes, and any complications. Descriptive statistics were used for continuous and categorial variables. If a patient had EBVs placed over multiple days, the duration from EBV placement was calculated from the day the first EBV was placed. Thoracostomy tube duration after EBV placement was calculated from the date of first EBV placement to the day the last thoracostomy tube was removed. The STROBE cohort reporting guidelines were used to report this study.^26^

## Results

During the study timeframe, there were six patients with necrotizing pneumonia who required venovenous ECMO during their hospitalization and underwent placement of one or more EBVs for severe air leak (Table 1). Five (83%) of the patients were female and the median age was 12 years, ranging from 18 months to 18 years. Five of the patients had no predisposing risk factors for lower respiratory infections, while one had B-cell acute lymphoblastic leukemia on maintenance chemotherapy with severe neutropenia at the time of presentation. All six patients were diagnosed with necrotizing pneumonia and acute respiratory distress syndrome. Five had viral pathogens detected, including two with influenza A, and one each with rhinovirus, influenza B, and SARS-CoV-2. Four had bacterial co-infections, including *Streptococcus pneumoniae* (3) and methicillin sensitive *Staphylococcus aureus* (1). Three patients were additionally diagnosed with septic shock, three with bacteremia, three with pulmonary hemorrhage, and two with multiorgan failure.

**Table 1.** Patient characteristics.

Patient	Age (years)/Sex)	Wt. (kg)	Co-morbidities	Diagnoses	Organism(s) identified
1	5, F	29	None	Necrotizing pneumonia, ARDS	Rhinovirus
2	18, F	53	B-cell ALL	Necrotizing pneumonia, ARDS, pulmonary hemorrhage, bacteremia, septic shock	MSSA
3	14, F	43	None	Necrotizing pneumonia, ARDS, bacteremia	Influenza A, *Streptococcus pneumoniae*
4	1yr 8mo, F	15	None	Necrotizing pneumonia, ARDS, bacteremia, atypical HUS	SARS-CoV-2, *Streptococcus pneumoniae*
5	16, F	75	None	Necrotizing pneumonia, ARDS, pulmonary hemorrhage, septic shock, multiorgan failure	Influenza A, MSSA
6	10, M	51	None	Necrotizing pneumonia, ARDS, pulmonary hemorrhage, septic shock, multiorgan failure	Influenza B, *Streptococcus pneumoniae*

ARDS = acute respiratory distress syndrome; ALL = acute lymphoblastic leukemia; MSSA = methicillin sensitive staphylococcus aureus; HUS = hemolytic uremic syndrome.

**Figure 1.**
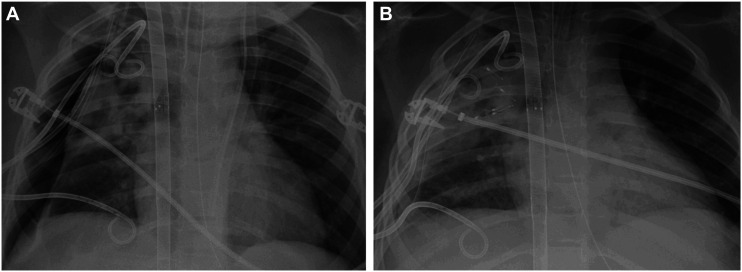
(a) Chest X-ray demonstrating persistent pneumothorax in a mechanically ventilated patient on venovenous ECMO with 3 right-sided chest tubes. (b) Same patient after placement of endobronchial valves.

The median duration of air leak prior to EBV placement was 12.5 days (range 1 to 29 days). The median number of endobronchial valves placed per patient was five, ranging from one to eleven (Table 2). Chest X-rays before and after EBV placement for one patient are shown in Figure 1. Three patients’ valves were placed through a laryngeal mask airway and two through a 7.0 mm endotracheal tube. One patient required the side-by-side approach, in which a 2.2 mm bronchoscope was used alongside the 2.6 mm deployment catheter through a 5.0 mm endotracheal tube and the valve was directed into the right middle lobe bronchus. Three patients required additional EBVs or replacement of some of their EBVs due to new air leak or recurrence of air leak identified in a region of the lung previously isolated by an EBV. Five of the patients had EBVs placed while they were on ECMO, and excluding the one death, the median number of days on ECMO after placement of the EBVs for these patients was 3.5 days (range 2 to 7 days). The median total duration of ECMO was 10 days (range 3 to 24 days), and the median number of days with a thoracostomy tube after EBV placement was 16 days (range 13 to 87 days).

**Table 2.** Hospitalization and valve placement characteristics.

Patient	Number of EBVs	EBV locations (sizes, mm)	EBVs placed while on ECMO	ECMO duration after EBVs (days)	Total ECMO duration (days)	Thoracostomy tube duration after EBVs (days)	Total EBV duration (days)	Disposition
1	5	RUL (5, 6)	Yes	2	7	16	114	Home
2	11	LUL, lingula, LLL, RUL (5, 6, 7, 9)	Yes	7	24	87	154	Inpatient Rehabilitation
3	6	LUL, lingula (5, 7, 9)	Yes	2	3	13	219	Inpatient Rehabilitation
4	1	RUL (5)	Yes	5	11	15	92	Inpatient Rehabilitation
5	5	LUL, lingula (6, 7)	Yes	16^a^	35^a^	16^a^	16^a^	Deceased
6	1	RML (5)	No	N/A	10	33	106	Inpatient Rehabilitation

EBV = endobronchial valve; ECMO = extracorporeal membrane oxygenation (all veno-venous); LOS = length of stay; NA = not applicable.

RUL = right upper lobe; LUL = left upper lobe; LLL = left lower lobe; RML = right middle lobe.

^a^Patient 5 died of multiorgan failure while on ECMO, calculated values represented until time of death.

One patient with bilateral multifocal cystic airspace disease developed a contralateral pneumothorax 24 hours after placement of the initial EBVs, which led to worsening respiratory failure, new thoracostomy tube placement, and cannulation onto venovenous ECMO. The patient underwent placement of new contralateral EBVs with improved air leak control. There was still an intermittent air leak for several months, so one-way Heimlich valves were temporarily used to promote mobility and rehabilitation until the air leak completely resolved. There was one death due to progressive multiorgan failure, which preceded EBV placement, and included thrombocytopenia associated multiorgan failure, refractory vasoplegia, and pulmonary hemorrhage. There was no significant bleeding reported in any patient as a direct result of EBV placement.

The total median duration of pneumonia-directed antibiotic coverage was 47 days (range 24 to 126 days). This includes a median of 20 days (range 11 to 29 days) of antibiotics prior to any EBV placement and a median of 15 days (range 6 to 89 days) after the last EBV placement in any patient. One patient developed fever and respiratory failure after an initial period of improvement with new infiltrate in lobes not treated with EBVs. Two patients developed respiratory symptoms after a period of recovery with new bacterial isolates from respiratory cultures (*Klebsiella pneumoniae*, *Stenotrophomonas maltophilia*) that were identified after first EBV placement. Both cases required extended antibiotic durations (30 and 60 days). One patient developed a new *Pseudomonas aeruginosa* urinary tract infection and had concurrent respiratory culture growth, but did not have clinical signs of pneumonia and antibiotic therapy was intended to target the urinary tract infection. None of the surviving patients had clinical signs of infection that were refractory to antibiotics, and prolonged antibiotic duration was not a deciding factor for timing of EBV removal.

Three (50%) patients underwent tracheostomy tube placement. Ultimately, one patient was discharged home and the remaining four were discharged to an acute rehabilitation center prior to home discharge. Excluding the one mortality, the median total length of stay was 75 days (range 50 to 179 days). All three patients with tracheostomies were weaned from the ventilator and had their tracheostomy tube eventually removed. All five surviving patients have had their EBVs removed with median EBV duration of 114 days (Table 2). Two patients required instillation of oxymetazoline at the time of removal for mild mucosal bleeding. One patient had a small residual hydropneumothorax that remained stable following valve removal and during follow up. There were no recurrences of air leak surrounding EBV removal.

## Discussion

We present the first series of critically ill pediatric patients who required ECMO for necrotizing pneumonia with severe air leak and underwent EBV placement. Five of the patients recovered, had their EBVs removed without air leak recurrence, and are at home off all respiratory support. One patient died from multiorgan failure which preceded EBV placement. This case series highlights the feasibility of using EBVs in acutely ill children with severe air leaks, including those on ECMO, which may expedite de-escalation of intensive care therapies.

Although EBVs were initially developed for adults with severe emphysema and persistent postoperative air leak, they have also been used in adult patients with air leak on ECMO to help wean from mechanical ventilation and ECMO.^17^ Recently, EBVs have been adapted for use in pediatric patients in variable clinical circumstances including necrotizing pneumonia with air leak, pulmonary hemorrhage, and postoperative air leaks.^20–24,27^ There have been only a few reported cases to-date across different institutions for which pediatric patients on ECMO had an EBV placed.^5,23,28,29^

Endobronchial valves show significant promise as acute interventions that may expedite recovery in critically ill pediatric patients with severe air leak. They are minimally invasive and present an alternative to invasive surgical procedures and irreversible pleural interventions. Conservative therapy, such as long-term thoracostomy tube placement may be preferred for stable patients. However, interventions that may hasten separation from mechanical ventilation and ECMO should be considered in certain patients who are exposed to those additional risks and complications, as prior literature has demonstrated that ECMO duration is independently associated with mortality.^6^ Timing and indication for EBV placement is not straightforward. In our cohort, patients were chosen based on a multidisciplinary discussion between pulmonology, critical care, anesthesiology, and general surgery. Generally, if an air leak was thought to be interfering significantly with ventilation and oxygenation, thus contributing to the need for ECMO, then a valve was discussed and placed.

Ultimately, only one of our six patients with necrotizing pneumonia and severe air leak requiring ECMO died (17%), which is lower than what has been described among children with severe pneumonia requiring ECMO (39%).^6^ Our experience suggests EBV placement in this patient population may expedite freedom from mechanical ventilatory and extracorporeal support. After EBV placement, two patients were decannulated from ECMO within 48 hours, the other two patients at 5 and 7 days. The two patients who did not undergo tracheostomy were extubated at 1 week and 2 weeks respectively after EBV placement.

Despite the success of these patients, EBVs must be used thoughtfully. One patient developed contralateral pneumothorax and new air leak within 24 hours of EBV placement. Although ipsilateral pneumothorax complication is described in EBV insertion for bronchoscopic lung volume reduction, a contralateral pneumothorax after valve insertion in a patient with ARDS and multifocal necrotizing pneumonia may be unrelated, but this requires further study.^30,31^ Fortunately, contralateral air leak was controlled by repeat bronchoscopy and EBV placement, allowing the patient to wean off ECMO shortly thereafter. Two other patients required repeat bronchoscopy and replacement of their valves for recurrent air leak in the same lobe, which were found to have either migrated or become malpositioned with loss of seal on repeat bronchoscopy. This was likely due to a combination of changes in the airway size and structure throughout the disease progression and migration of the valves, or inappropriate size selection during initial placement. This was resolved with upsizing or repositioning the existing EBVs.

Antibiotic duration is another challenging topic for both necrotizing pneumonia and in patients with EBV placement. Post-obstructive pneumonia developed in up to 13% of adult patients who had EBVs placed for emphysema.^30,31^ It is unknown whether this risk is higher in patients still being treated for underlying necrotizing pneumonia. Our patients received prolonged courses of antibiotics, with two patients that developed clinical signs of respiratory infection after first EBV placement with new respiratory bacterial isolates that prompted prolonged antibiotic courses. It is unclear whether either of these were related to EBV placement, or if this is further evidence of high rates of nosocomial infections in critically ill children.^32^ However, all five surviving patients were successfully treated and have had no recurrence of pneumonia after the antibiotic courses associated with their initial necrotizing pneumonia and critical illness course through EBV removal. Additionally, the placement of endobronchial valves to potentially facilitate liberation from high-risk therapies like extracorporeal support must be balanced with the potential need for extended antibiotic courses. The duration of antimicrobial therapy for patients with severe air leak from necrotizing pneumonia with EBV placement is unclear. Our experience suggests prolonged antibiotic durations may be beneficial and vigilance about infectious complications during the period of critical illness is necessary.

Additionally, EBVs pose technical challenges. The deployment catheters for the EBVs available in the USA require a 2.6 mm working channel in a flexible bronchoscope, limiting its use to large bronchoscopes. This would have made EBV placement in several of our patients impossible via an endotracheal tube. While supported on ECMO, these patients’ artificial airways were safely and transiently switched to a laryngeal mask airway, which facilitated a larger flexible bronchoscope and successful EBV placement. In essence, this made ECMO an important facilitator for EBV placement as much as it was an indication. This required careful coordination between pulmonology, critical care, anesthesiology, respiratory therapy, nursing, and ECMO specialists. In the one patient who developed an air leak after ECMO decannulation, the 5.5 mm endotracheal tube was not large enough to facilitate traditional placement of an EBV. Instead, the deployment catheter was inserted parallel to a 2.2 mm flexible bronchoscope, which was used to direct the valve into the right middle lobe. However, we were not able to place a valve in the patient’s lingula where an additional air leak was isolated. A smaller deployment catheter that requires only a 2.0 mm working channel is available in Europe and would likely improve the feasibility of valve placement in children.

Finally, the timing of EBV removal for this indication is uncertain. The median duration in our cohort was 114 days, which is significantly longer than the 6 weeks recommended by the manufacturer when used for postoperative air leak. However, endobronchial valves have been left in much longer for bronchoscopic lung volume reduction.^11,33^ The prolonged duration in our cohort was mainly dictated by our cohort’s prolonged recovery time, which included hospitalization, rehabilitation, separation from long-term ventilatory support, and lung recovery.

### Study limitations

Our retrospective case series is limited by the small sample size and significant heterogeneity of patients, which limits its generalizability. All six patients had severe air leaks in the setting of necrotizing pneumonia that required ECMO during their hospitalization, but the decision to proceed with EBV placement was made through a multidisciplinary discussion between pediatric interventional pulmonologists, surgeons, and critical care physicians. There are many unknowns with using EBVs in children, including the timing of EBV removal, complication rates, and optimal antibiotic duration. Finally, we cannot definitively state that EBV placement expedited de-escalation of intensive care without a controlled study.

## Conclusion

Endobronchial valves are a feasible intervention for severe air leak in critically ill children with necrotizing pneumonia and may expedite liberation from extracorporeal support. Prospective studies are warranted to determine the efficacy and safety of EBV usage in this complex patient population relative to traditional management strategies.

## Appendix

### Abbreviations

ALL: Acute lymphoblastic leukemia
ARDS: Acute respiratory distress syndrome
EBV: Endobronchial valve
ECMO: Extracorporeal membrane oxygenation
LLL: Left lower lobe
LOS: Length of stay
LUL: Left upper lobe
MSSA: Methicillin sensitive *Staphylococcus aureus*
NA: Not applicable
PICU: Pediatric intensive care unit
RUL: Right upper lobe.
